# Determinants of unmet physical and psychological supportive care needs among adult cancer patients in Southern Ethiopia

**DOI:** 10.1136/spcare-2023-004606

**Published:** 2023-10-10

**Authors:** Asaye Amenu, Amdehiwot Aynalem, Yacob Abraham Borie, Wegene Jemebere, Ezedin Molla, Beniyam Samuel, Eskinder Israel, Tomas Yeheyis, Dawit Getachew Assefa, Meless Gebrie

**Affiliations:** 1 School of Nursing, College of Medicine and Health Sciences, Hawassa University, Hawassa, Ethiopia; 2 Department of Midwifery, College of Health Sciences and Medicine, Dilla University, Dilla, Ethiopia; 3 School of Public health, College of Medicine and Health Sciences, Wolaita Sodo University, Wolaita Sodo, Ethiopia; 4 Department of Nursing, College of Health Sciences and Medicine, Dilla University, Dilla, Ethiopia

**Keywords:** Supportive care

## Abstract

**Objective:**

The main objective of this study was to assess the prevalence of unmet physical and psychological supportive care needs and associated factors among adult patients with cancer in Southern Ethiopia.

**Methods:**

A cross-sectional study was conducted among 321 patients with cancer from 20 June 2022 to 5 August 2022 at Hawassa University Comprehensive Specialized Hospital oncology centre. Simple random sampling technique was used to recruit participants. Data were entered into Epi-Data V.4.6 and were exported to SPSS V.26 for analysis. Logistic regression model was used to describe the association between dependent and independent variables.

**Result:**

The mean age of the study participants was 45±14.27. The prevalence of unmet physical and psychological supportive care needs was 47.3% and 71.1%, respectively. Rural residence ((adjusted OR, AOR 2.73; 95% CI (1.27 to 5.83)) and late-stage cancer ((AOR 2.95; 95% CI 1.02 to 8.52) were factors significantly associated with unmet physical supportive care need. Coexisting illness was associated with both unmet physical and psychological supportive care needs (AOR 2.73; 95% CI 1.27 to 5.83) and (AOR 2.71; 95% CI 1.16 to 6.33), respectively.

**Conclusion:**

Nearly half of the study participants had an unmet physical supportive care need while greater than two-thirds had unmet psychological supportive care need. Residence and late-stage cancer were factors significantly associated with physical unmet supportive care need while coexisting illness was associated with both unmet physical and psychological supportive care needs. Hence, supportive care for patients with cancer should be given an emphasis and incorporated into the cancer treatment protocol.

**WHAT IS ALREADY KNOWN ON THIS TOPIC**

- Unmet need regarding healthcare services refers to the gap between a people’s desire or need for those services and the actual experience of receiving them.
- Unmet physical and psychological supportive care needs lead patients with cancer to ineffective coping, worsened emotional distress and a reduced quality of life.

**WHAT THIS STUDY ADDS**

- This study revealed the prevalence of unmet physical and psychological supportive care needs among adult patients with cancer and associated factors adding the relevant factors affecting the physical and psychological needs of adult patients with cancer.
- The study adds which areas need an attention to meet the supportive care needs of adult patients with cancers.

**HOW THIS STUDY MIGHT AFFECT RESEARCH, PRACTICE OR POLICY**

- The study will enable evidence-based policy-making targeted at improving supportive care for patients with cancer and help healthcare practice improvement by informing the extent of the problem.
- It will be used as an input for other studies to be conducted by other interested researchers on similar and related topics.

## Introduction

 Supportive care is a person-centred approach which includes provision of a variety of services for those living with or affected by cancer to meet their informational, spiritual, emotional, social or physical needs during diagnosis, treatment or follow-up phases and basically involves promotion and prevention, survivorship, palliation and bereavement.^1^

Unmet need regarding healthcare services refers to the gap between a people’s desire or need for those services and the actual experience of receiving them. These needs can occur at any stage in the disease course, from diagnosis to the completion of treatment or death.^2^ It has been categorised into five major domains: physical, psychological, informational, patient care and sexual needs.^3^ Various researchers have found that the greatest unmet needs are associated with psychological needs.^4^

The findings from various studies conducted among the general cancer population reported that the low to high level of unmet patient supportive care needs (SCNs) ranged 27%–60.2%.^5^,^7^ Also, similar results reported from the study conducted in the UK.^5^ Based on the survey conducted in the Africa region, approximately 46% of the study respondents reported unmet SCNs.^8^

In addition to the impact of medications and other therapies on the effectiveness of cancer treatment, the level of unmet needs can adversely affect the outcome of cancer treatment.^9^ Studies have shown that unmet SCNs lead to ineffective coping, worsened emotional distress and a reduced quality of life.^10 11^

Several factors were identified affecting the unmet SCNs and those factors are the type of cancer, stage of the disease and the impact of specific sociodemographic factors such as age, gender, marital status, income level, occupation, lengthy cancer experience and anticancer treatments need.^12 13^

Assessing the unmet SCNs of patients with cancer has a lot of benefits for both the patient and the government. It helps to prioritise services, allocate resources based on the urgency of need, identify a subset of patients with higher preventive needs or at least to reduce problems through appropriate early intervention.^13^

So far, the Ethiopian government has made significant efforts to improve the supportive patient care by employing the Ethiopian National Cancer Control Plan (2016–2020). In addition, the Ethiopian Ministry of Health established five specialised cancer treatment centres in Hawassa, Gondar and Jimma. In addition, there is little evidence in Ethiopia particularly in this study area about the supportive physical and psychological care need among patients with cancer. Therefore, this study was aimed to assess the prevalence of unmet SCN and its associated factors among patients with cancer at Hawassa University Comprehensive Specialized Hospital (HUCSH) oncology centre.

## Materials and methods

### Study design

An institution-based quantitative cross-sectional study was conducted.

### Study area and period

The study was conducted at HUCSH oncology centre in Hawassa city, Southern Ethiopia. Hawassa city is located 275 km south of Addis Ababa. HUCSH oncology centre has a total of 780 and 63 new joining patients per year and month, respectively. Around 240 patients visit the centre for the chemotherapy, and 140–150 patients have follow-up in a month. In this oncology centre, there are 15 nurses, 3 general practitioners and 2 oncologists. The study was conducted from 20 June 2022 to 5 August 2022.

### Population

#### Source population:

All patients with cancer at HUCSH oncology centre.

#### Study population:

A randomly selected patients with cancer aged ≥18 years at HUCSH oncology centre during the data collection period.

### Inclusion and exclusion criteria

#### Inclusion criteria

Sampled patients with cancer aged ≥18 years at HUCSH oncology centre who were volunteer and consented to participate during the data collection period were included.

#### Exclusion criteria

Patients who were unable to communicate and critically ill during the data collection period were excluded.

### Sample size determination and sampling procedure

#### Sample size determination

The sample size was calculated using a single population proportion formula with 5% marginal error (d) and CI of 95% (Z α/2=1.96) based on the estimated proportion of unmet physical SCN (74.6%) from the study conducted at Dessie Referral Hospital, Dessie, South Wollo, North East Ethiopia.^14^

n= (Z α/2)^2^ P (1−P)/d2.

Based on these assumptions:

no=is the total sample size required.

Zα/2 is the CI at 95% (1.96).

P is the estimated proportion.

d=is the margin of error which is 5% (0.05).

Where: n=sample size required.

no=sample obtained from single population formula.

n = [1.96]^2^×0.746 (1–0.746)/(0.05)2=291.

By adding 10% non-response rate the final sample size was 321.

#### Sampling technique and procedure

A simple random sampling method was used to select 321 study participants from the total 390patients with cancer in the oncology unit who were available during the study period.

## Variables

### Dependent variables

Unmet physical and psychological SCN.

### Independent variables

#### Sociodemographic characteristics

Age, sex, educational status, marital status, education, occupation.

#### Disease-related factors

Type of cancer, type of treatment, time since diagnosis, stage of cancer, symptoms of cancer, duration since treatments started, types of treatments, side effects of the treatment.

#### Information-related factors

Sources of information and informational status about the disease, diagnosis, treatment and follow-up.

### Data collection instruments

An interviewer-administered validated and pretested questionnaire was used to collect data from the study participants. Needs assessments were completed with the Supportive Care Needs Survey (SCNS) tool, which is validated and consisting of 25 items (Amane et al, 2021). The participants were interviewed by using structured questionnaire adapted from different literatures and SCNS tool to indicate the level of their needs for the last month based on a 5-point Likert scale. The need for help was rated on a 5-point scale as follows: 1=not applicable, 2=satisfied, 3=low need, 4=moderate need and 5=high need. Individual need items were dichotomised into no need (not applicable-to-satisfied) and some need (low-to-high needs) to assess the proportion of patients reporting individual unmet needs.^4^

### Data quality control measures

One-day training was given for supervisors and data collectors on the objective and significance of the study, how to collect and record the appropriate information, procedures of data collection techniques, the whole contents of the questionnaire and to keep confidentiality. A pretest was done on 5%^13^ of the samples before the actual survey and important modifications were made based on the findings. Data were collected by four BSc nurses and supervised by two supervisors. During the data collection time, close supervision and monitoring were done every day by the supervisors to ensure the quality of the data. At the end of each day, the collected data were checked for completeness and logical consistency.

### Data analysis

The collected data were cleaned, coded and entered into Epi-Data V. 4.6 and exported to SPSS V.26 for analysis. Descriptive statistics such as frequencies and proportions were used to summarise the data. Bivariate and multivariate analyses were used to examine the relationship between the outcome variables and independent variables. Variables with p value less than 0.25 during binary logistic regression were entered into the multivariable logistic regression. Adjusted ORs (AOR) and their 95% CI were used as indicators of the strength of association. Statistical significance was set at p values of less than 0.05.

### Operational definition

Items were dichotomised into no need (not applicable-to satisfied) and some need (low-to-high need).

The outcome variable was recoded into ‘no needs’ and ‘some needs. ‘Unmet needs’ If a patient is reported as having at least one low to high need in a domain, and ‘no needs’ if a patient reports no need in all items in a single domain.

The five response options are described as follows:

1. Not applicable: it was not a problem for the patient as a result of having cancer.
2. Satisfied: he did need help with this, but his need for help was satisfied at the time.
3. Low need: little concern or discomfort.
4. Moderate level: some concern or discomfort.
5. High need level: a lot of concern or discomfort. Individual unmet needs are reported by a high proportion of patients.^4^

## Result

### Sociodemographic characteristics of participant

Out of 321 sampled patients with cancer, 311 participants were involved in this study giving a response rate of 96.9%. The mean age of the respondents was 45±14.27 years, with a minimum age of 18 and a maximum of 76 years. Out of the total respondents, 193 (62.1%) were female. One hundred and eighty-eight (60.5%) of the participants came from urban areas. Among participants, 133 (42.4) had the lowest monthly income (table 1).

**Table 1:** Sociodemographic characteristic of patients with cancer in HUCSH, Hawassa, Ethiopia, 2022 (n=311)

Variables	Category	No of participants (n)	%
Sex of the patient	Male	118	37.9
	Female	193	62.1
Education status of participants	Unable to read and write	33	10.6
	Primary completed	73	23.5
	Secondary completed	104	33.4
	Certificate and diploma	25	8
	Degree and above	76	24.4
Occupational status	Employed	51	16.4
	Private work	175	56.3
	Jobless	82	26.4
	Retired	3	1
Marital status	Single	42	13.3
	Married	238	76.5
	Divorced	19	6.1
	Widowed	12	3.9
Residence	Urban	188	60.5
	Rural	123	39.5
Income	Lowest	133	42.4
	Secondary lowest	65	20.9
	Middle	36	11.6
	Secondary highest	51	16.4
	Highest	27	8.7

HUCSHHawassa University Comprehensive Specialized Hospital

### Clinical characteristics of participant

One hundred and fifteen (37%) of the study participants had breast cancer, followed by digestive system cancer cases 94 (30.2%). Most of the patient (86.5%) chemotherapy was found to be the leading treatment given to patients with cancer; it accounts for of the total, and the majority of participants had stage IV cancer. Among participants, 67 (21.5%) had a history of remission and 159 (51.1%) had a probability of recurrence. Thirty-six (11.6%) of participants had a history of coexisting illnesses (table 2).

**Table 2:** Clinical characteristics of oncological patient at HUCSH, Hawassa, Ethiopia, 2022 (n=311)

Variables	Category	No of participants (n)	%
First cancer site	Breast	115	37
	Colon and rectum	94	30.2
	Prostate	8	2.2
	Lung	22	7.1
	Skin	10	3.2
	Cervical	20	6.4
	Lipoma	22	7.1
	Unknown	7	2.3
	Other	13	4.2
Treatment options of the patients	Chemotherapy	239	76.8
	Radiotherapy	3	1
	Surgery	32	10.2
	Hormonal treatment	6	1.9
	Analgesia	31	10
Stage of cancer	Stage I	10	3.5
	Stage II	41	21.2
	Stage IIIStage IV	107122	40.035.3
	Unknown	31	10
Remission of the disease	YesNo	67244	21.578.5
Recurrence of the disease	YesNo	159152	51.148.9
Coexisting disease	YesNo	36275	11.688.4

* N.B; co-existing illnesses: RVI, DM, HTN.

DMDiabetes MellitusHTNHypertensionHUCSHHawassa University Comprehensive Specialized HospitalRVIRetroviral infection

### Information status

From a total of 311 participants, 257 (82.6%) were getting information about their diagnosis. One hundred and sixty-two (52.1%) participants were informed about their current status of a disease. Among participants, only 61 (19.6%) were informed about their possible cause of the disease. From 311 participants, 140 (45%) and 149 (47.9%) were informed about their medical tests and medical results, respectively. One hundred and eighty-five (59.5%) patients claimed that they were informed about medical treatment they were taking (table 3).

**Table 3:** Informational status of oncological patient at HUCSH, Hawassa, Ethiopia, 2022 (n=311)

Variables	Category	No of participants (n)	%
Informed about diagnosis	YesNo	25754	82.617.4
Informed about spread/stage/current status of the disease	YesNo	162149	52.147.9
Informed about possible causes for your disease	YesNo	61250	19.680.4
Informed about medical tests you undergo	YesNo	140171	4555
Informed about medical results you have received	YesNo	149162	47.952.1
Informed about medical treatments	YesNo	185126	59.540.5
Informed about sequence of medical treatments	YesNo	167144	53.746.3
Informed about expected benefit of the treatment	YesNo	186125	59.840.2
Informed about possible side effect of treatment	YesNo	196115	6337
Informed about duration of treatment	YesNo	188123	60.539.5
Source of information			
Health professionalHealth professional and readingFrom the other patients	2059412		65.930.23.9

HUCSHHawassa University Comprehensive Specialized Hospital

### The proportion of the **SCNs** of participants

The prevalence of unmet physical and psychological SCN was 47.3% and 71.1%, respectively.

### Top ten most frequent needs reported by participants

**Figure 1:**
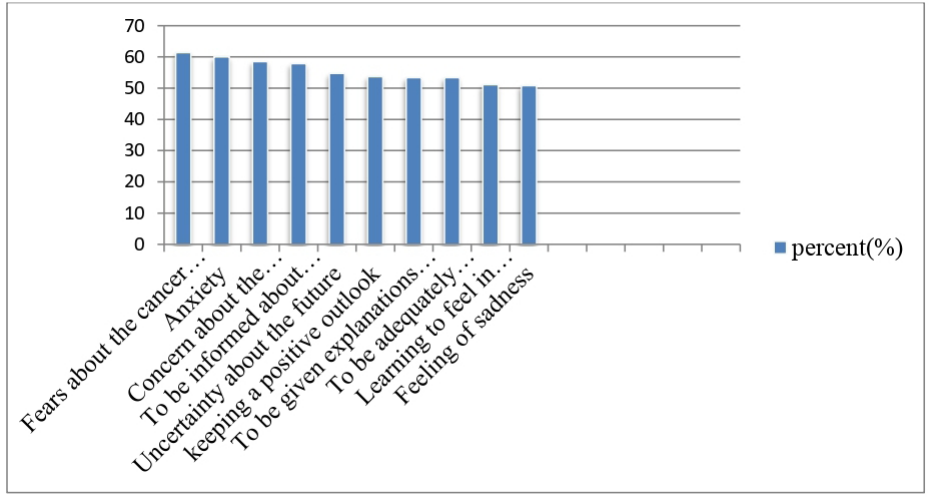
Top 10 unmet supportive care need item, Hawassa University Comprehensive Specialized Hospital, Hawassa, Ethiopia, 2022.

Top 10 unmet needs reported by patients with cancer were fear about cancer spreading (61.4%), followed by anxiety (60%), concern about the (58.5%), to be informed about the test results (57.9%), uncertainty about the future (54.7%), keeping the positive outlook (53.7%), to be given explanations of those tests (53.4%), to be adequately informed about the benefits (53.4%), learning to feel in control (51.1%) and feeling of sadness (50.8%), respectively (figure 1).

### Factors associated with physical/daily living domain

Binary logistic regression analysis revealed that marital status, residence, stage of cancer, remission status, coexisting illness, information about the sequence of medical treatment, information about expected benefits of treatment and having information about the duration of treatment were candidate variables for the final multivariable model. However, residence, stage of cancer and coexisting illness were significant factors in the multivariable logistic regression model.

Patients from rural areas were nearly seven times more likely to have unmet physical SCN than patients from urban areas (AOR 6.75; 95% CI 3.91 to 11.67). Similarly, the odds of unmet physical SCN were approximately three times higher in patients with late-stage cancer (AOR 2.95; 95% CI 1.02 to 8.52) as compared with their counterparts. Moreover, the odds of unmet physical SCN were approximately three times higher in patients with coexisting illness (AOR 2.73; 95% CI 1.27 to 5.83) as compared with those who had no coexisting illness (table 4).

**Table 4:** Bivariable and multivariable analysis of physical domain among oncological patient in HUCSH, Ethiopia, 2022

Variable	Physical domain		COR	AOR with 95% CI	P value
	Some need (%)	No need (%)			
Marital status					
Single	14 (33.30)	28 (66.70)	1	1	
Married	120 (50.40)	118 (49.60)	2.03	1.67 (0.75 to 3.69)	0.204
Divorced	10 (52.60)	9 (47.40)	2.22	2.41 (0.68 to 8.48)	0.169
Widowed	3 (25.00)	9 (75.00)	0.66	0.43 (0.08 to 2.22)	0.316
Residence					
Rural	91 (74.00)	32 (26.00)	6.7	6.75 (3.91 to 11.67)	0.001**
Urban	56 (29.80)	132 (70.20)	1	1	
Cancer stage					
Early	26 (51.00)	25 (49.00)	1.89	2.07 (0.86 to 4.99)	0.102
Late	110 (48.00)	119 (52.00)	1.68	2.95 (1.02 to 8.52)	0.045*
Unknown	11 (35.50)	20 (64.50)	1	1	
Remission status					
Yes	41 (61.20)	26 (38.80)	2.05	1.62 (0.85 to 3.06)	0.138
No	106 (43.40)	138 (56.60)	1	1	
Co-existing illness					
Yes	34 (69.40)	15 (30.6)	2.98	2.73 (1.27 to 5.83)	0.009*
No	113 (43.10)	149 (56.90)	1	1	1
Information about sequence of medical treatment					
Yes	84 (50.30)	83 (49.70)	1.3	0.89 (0.48 to 1.63)	0.713
No	63 (43.80)	81 (56.3)	1	1	
Information about expected benefits of treatment					
Yes	94 (50.50)	92 (49.50)	1	1	
No	53 (42.40)	72 (57.60)	1.38	1.01 (0.52 to 1.93)	0.976
Have information about duration of treatment					
Yes	94 (50.00)	94 (50.00)	1.32	1.46 (0.81 to 2.65)	0.202
No	53 (43.10)	70 (56.90)	1	1	

* Indicates significance.

AORadjusted ORHUCSHHawassa University Comprehensive Specialized Hospital

### Factors associated with psychological domain

Binary logistic regression analysis revealed that educational status, house hold income, coexisting illness and informational status were candidate variables for the final multivariable model. Coexisting illness was a significant factor in the multivariable logistic regression model.

The odds of unmet psychological SCNs were approximately three times more in patients with coexisting illness (AOR 2.71; 95% CI 1.16 to 6.33) as compared with those who have not coexisting illness (table 5).

**Table 5:** Bivariable and multivariable analysis of psychological domain

Variable	Psychological domain		COR	AOR (95% CI)	P value
	Some need (%)	No need (%)			
Education status					
Unable to write and read	24 (72.70)	9 (27.30)	0.95	0.78 (0.28 to 2.15)	0.641
Primary education	45 (61.60)	28 (38.40)	0.57	0.51 (0.24 to 1.10)	0.089
Secondary education	81 (77.90)	23 (22.10)	0.25	1.39 (0.66 to 2.93)	0.381
Certificate and diploma	15 (60.00)	10 (40.00)	0.53	0.60 (0.22 to 1.60)	0.309
Degree and above	56 (73.70)	20 (26.30)	1	1	
Wealth rank					
Lowest	59 (81.90)	13 (18.10)	1.79	1.63 (0.67 to 3.98)	0.280
Second lowest	46 (63.00)	27 (37.00)	0.67	0.67 (0.29 to 1.50)	0.332
Middle	26 (70.30)	11 (29.70)	0.93	1.09 (0.40 to 2.91)	0.860
Second highest	47 (68.10)	22 (31.9)	0.84	0.79 (0.35 to 1.76)	0.568
Highest	43 (71.70)	17 (28.30)	1	1	
Co-existing illness					
Yes	41 (83.70)	8 (16.30)	2.33	2.71 (1.16 to 6.33)	0.020*
No	180 (68.70)	82 (31.30)	1	1	
Diagnosis					
Yes	176 (68.50)	81 (31.50)	1	1	
No	45 (83.30)	9 (16.70)	2.30	2.14 (0.96 to 4.77)	0.062
Informed about the possible cause of disease					
Yes	38 (62.30%)	23 (37.70)	0.60	0.65 (0.34 to 1.23)	0.186
No	183 (73.20)	67 (26.80)	1	1	
Information about the possible side effect of treatment					
Yes	133 (67.90)	63 (32.10)	0.64	0.72 (0.41 to 1.28)	0.275
No	88 (76.50)	27 (23.50)	1	1	

* Indicates significance.

AORadjusted ORCORcrude odds ratio

## Discussion

The current study revealed the prevalence of unmet physical and psychological SCN and its determinants among patients with cancer admitted at HUCSH oncology centre.

The evaluation of patient needs and satisfaction has grown to be a crucial and essential component of the healthcare system. It is imperative to assess patient satisfaction in order to know whether their needs were addressed. A care programme must address the perceived SCNs of patients with cancer. The accurate identification of cancer patients’ SCNs is the initial step in planning and intervening for supportive care.

In various healthcare systems, the two main factors used to assess the quality of health services are patient demands and satisfaction. Cancer patients’ unmet requirements and their satisfaction with their overall care were found to have an impact on health-related quality of life. As a result, it is advised to address the unmet requirements of patients with cancer and make sure there is a better satisfaction rate in order to maintain a sufficient level of health-related quality of life^15 16^

In this study, the prevalence of unmet needs for supportive care in physical and psychological need was 47.3% and 71.1%, respectively. Our analysis indicates that there is a significant unmet need for psychological supportive treatment. This is because psychological issues were more prevalent in patients with cancer. Cancer diagnoses, treatments and their negative impact on patients’ emotions and thoughts are the primary causes of psychological issues.^17^ According to another study, patients with cancer frequently suffered with the emotional effects of their sickness and a sense of powerlessness, and they required emotional, financial, spiritual and informational support during all phases of treatment.^18^

This study finding is similar to studies conducted in Nigeria, the UK, Australia and the United Arab Emirates.^5 8 19 20^ The possible reason might be due to low provision of psychological support for patients from the families, caregivers and healthcare professionals. However, it is higher than the studies conducted at Malaysia and Mexico in which 53.3% and 39.3% of the respondents at unmet psychological need respectively.^21 22^ A possible explanation might be the differences in cultural context of the study area.

This study found that 47.3% of study participants had unmet physical needs for supportive care, which is consistent with the findings from Lagos University Teaching Hospital in Nigeria, where 47.4% of study participants had unmet physical needs.^8^ But the finding is lower than the studies from Jakarta, Indonesia and Dessie Referral Hospital, Northern Ethiopia in which 81% and 80.4% had unmet physical demands, respectively.^10 14^ This may be due to the reason that there is low awareness of the physical needs in the study area. Contrarily, it is less the findings which are done in Malaysia and Mexico, where 38.6% and 19.1% of respondents, respectively, had unmet physical needs.^21 22^ This suggests that supportive care should be increased and/or given to lessen physical needs. For those who are affected by cancer and their families, the disease has severe physical, emotional, social and financial repercussions. Patients with cancer have also been found to experience a variety of issues, including fear of dying, pain, disability, infertility, dependency, abandonment, altered relationships and financial hardship.^11^

According to this study, the top 10 unmet SCNs were fear of cancer spreading (61.4%), anxiety (60%), worry about (58.5%), need to know test results (57.9%), uncertainty about the future (54.7%), maintaining a positive outlook (53.7%), receiving explanations of those tests (53.4%), need to be adequately informed about the benefits (53.4%), developing a sense of control (51.1%) and sadness (50.8%). This is consistent with a study done in Japan, which found that the fear of cancer spreading was the most common unmet SCNs.^17^ On the other hand, a study from Asia indicated that uncertainty about the future was the most common unmet supportive care demand.^22^

Our study discovered that the factors such as domicile or residence, cancer stage and coexisting illness were strongly associated with unmet physical and psychological SCN. Unmet physical SCNs were substantially correlated with place of residence and cancer stage. Coexisting illnesses were associated with both unmet need of physical and psychological supportive care.

Compared with individuals who lived in urban regions, participants who had been living in rural areas were more likely to have high levels of unmet physical SCNs. This result differs from one study conducted by a Gonder University, which found that patients from rural areas are less likely to have unmet needs for supportive care.^2^ This might be due to the difference in the study’s methodology and the participants socioeconomic status. However, given that patients in urban areas are more aware of and interested in their underlying conditions, thus this finding was unexpected and requires further research to clarify and confirm this association.

Significant levels of unmet physical supportive care demands were more prevalent among participants with late-stage cancer than among participants with early-stage cancer. This is in line with research from Dessie in the Amhara Region of Ethiopia, which found that patients with advanced cancer were twice as likely to have unmet physical, psychological and health information needs.^22^ Similar findings from a Malaysian study showed that cancer survivors who were diagnosed at an advanced stage had higher physical and psychological needs.^22^ This may be related to patients with advanced disease who will need complex and prolonged treatment, have unexpected side effects, and struggle with complex and lengthy treatment, leading them to have physical and psychological unmet needs. Also, patients in the late stages may have more perceived needs than those in the earlier stages.

The patients who have coexisting illness were more likely to have high levels of both physical and psychological SCN than those who have no coexisting illness and there is no research that reports the association between coexisting illness and both physical and psychological SCN. The possible explanation might be the patients who experienced comorbidities could show SCN compared with those patients who did not experience such illness.

## Conclusion and recommendation

According to our research, patients with cancer at the HUCSH oncology centre have a significant proportion of unmet needs for psychological supportive care, followed by needs for physical SCNs. Coexisting illness was the only predictor related to both physical and psychological SCNs, while patients from rural regions and those with late-stage cancer were factors strongly linked to unmet physical SCNs. In order to fulfil SCNs in accordance with patients’ perceived needs, an efficient psychological and physical care intervention is crucial.

It would be also preferable if researchers conduct a study that identifies the unmet SCNs of patients with cancer using a qualitative approach or mixed method.
